# Qualitative Bereavement Experiences and Support in Community-Dwelling Older Adults: A Scoping Review

**DOI:** 10.1177/00302228241308616

**Published:** 2024-12-15

**Authors:** Samantha Teichman, Barbara A. Mitchell, Indira Riadi, Habib Chaudhury, Albert Banerjee, William Odom

**Affiliations:** 1 Department of Gerontology, 1763 Simon Fraser University, Vancouver, British Columbia, Canada; 2 Department of Sociology & Anthropology, 1763 Simon Fraser University, Burnaby, British Columbia, Canada; 3 Department of Gerontology, 67112 St Thomas University, Fredericton, New Brunswick; 4 School of Interactive Arts and Technology, 1763 Simon Fraser University, Surrey, British Columbia, Canada

**Keywords:** older adults, grief, community dwelling, bereavement, coping

## Abstract

This scoping review examines qualitative studies on bereavement experiences and support mechanisms for community-dwelling older adults. This area of research is especially timely given rapid population aging and the interplay of heightened vulnerability, cumulative losses, shrinking support systems, and the critical need for bereavement support. Such support not only acts as a preventative health measure but can also serve as a catalyst for fostering meaning-making and navigating grief in later life. Through a scoping search and synthesis of relevant qualitative studies (*n* = 24), the findings reveal four key themes: the impact of grief on wellbeing, grief perceived as an individual issue, challenges in seeking and receiving support, and positive support experiences and transformations. Knowledge gaps, trends and issues, and areas for further investigation are also highlighted. This discussion contributes to developing targeted bereavement support strategies for community-dwelling older adults.

As we age, encounters with grief become more frequent (Treml et al., 2022; Zoler, 2006), necessitating a deeper understanding of the specific needs of older adults and the vital role of bereavement care in their wellbeing. Bereaved older adults face multiple challenges such as increased vulnerability (Treml et al., 2022; Zoler, 2006), health impacts (Guité-Verret et al., 2021; Ott et al., 2007), cumulative losses (Garrett, 1987), changes to support networks (Bankoff, 1983; Bellamy et al., 2014), and increased awareness of their mortality (Groves, 2010; Gruenewald et al., 2007). Bereavement care addresses immediate emotional impacts, and serves as a preventative health measure, mitigating psychological decline and promoting resilience. Older adults experiencing loss often describe experiences of isolation as they have relied heavily on the deceased for emotional support, companionship, and daily activities (Bankoff, 1983; Bennett, 2010). It is therefore critical to acknowledge that the process of grieving extends beyond the physical absence of the deceased, involving the mourning of roles, identities, and routines (DiGiacomo et al., 2015; Koren, 2016).

Unlike most of their younger counterparts, older adults will likely have experienced grief. The concept of continuing bonds (Klass et al., 1996) posits that our relationship with the deceased endures. While this continued relationship may welcome positive memories (Wilson et al., 2024), it is critical to recognize the challenges posed by unresolved grief (Field, 2006). The adaptiveness of continuing bonds in grief management depends on various factors, including how the bereaved perceives the bond, the quality of the predeath relationships, and the bereaved’s afterlife beliefs (Root & Exline, 2014). For those who have experienced cumulative grief over time, these dynamics are especially relevant as they may struggle to process multiple losses. Yet, there is still much to learn about how accumulated grief and grief triggers may evolve or persist as bonds are maintained. Relatedly, our social circles often shrink as we age (Carstensen, 1992), and the loss of someone from this limited network can intensify the need for support. Experiencing bereavement in later life often prompts confrontation with one’s own mortality, leading to existential reflection and the search for meaning (Groves, 2010; Neimeyer et al., 2014). Thus, for community-dwelling older adults, the intertwining of increased vulnerability, cumulative losses, changing support systems, and existential reflections underscores the vital role of bereavement care in preventing further decline in health and facilitating a meaningful exploration of life’s purpose in later years. While spousal loss (Davidow et al., 2022) and parental bereavement in older age (Wright, 2022) have been reviewed, broader bereavement experiences among community-dwelling older adults remain underexplored.

This scoping review thematically analyzes existing qualitative literature pertaining to bereaved community-dwelling older adults, focusing on their personal experiences, including the role of informal and formal support systems. The guiding research question is: *How do community-dwelling older adults experience bereavement, and what is the role of informal and formal support systems in shaping their grief and coping mechanisms?* Emphasizing the community context highlights gaps in bereavement resources distinct from institutional settings. This review focuses on qualitative and mixed methods studies that capture subjective, lived experiences through methods like interviews, narratives, and observations. These methods delve into the deeper, subjective aspects of grief, revealing the diverse ways individuals make sense of loss, find meaning, and cope, including the impact of various support types on their grief journey. Therefore, this review underscores the unique aspects of late-life bereavement in community settings, encompassing informal and formal support systems.

## Methods

### Search Strategy

This scoping review follows the Preferred Reporting Items for Systematic Reviews and Meta-Analyses extension for Scoping Reviews (Tricco et al., 2018), which builds on Arksey and O’Malley (2005) methodology, expanded on by Levac, Colquhoun, and O’Brien (2010). We began by defining the research question and objectives related to older adults’ bereavement experiences and establishing eligibility criteria. The second step involved developing a comprehensive search strategy, detailing databases, key terms, and search strings relevant to the research focus (see Table 1). After this step, we employed screening processes to select studies. We used a data charting form to extract relevant information, such as demographics, coping mechanisms, and support systems (see Table 2) to ensure transparency. Results were then synthesized thematically, highlighting patterns, challenges, and gaps, leading to a comprehensive summary of the research evidence, discussing implications for healthcare, social support, and future research.

**Table 1.** Search Strategy.

Search Terms	Older Adults	Community Settings	Bereavement	Qualitative Methods
	Older adult*, elderly, geriatric*, aging, senior*, aged 65, 65+	Community-dwelling*, community dwelling*, living at home, community setting*	Bereavement, grief, loss, mourning, death	Qualitative research, qualitative study, qualitative method*, interview*, mixed method*, multimethod*
Databases	AgeLine; PsychINFO; social sciences full text (H.W. Wilson); CINAHL; handsearching through google scholar			
Inclusion	Published in English; peer-reviewed; Academic journal; primary study; qualitative bereavement experiences; study participants are community-dwelling bereaved older adults; family member death; published after 2000			
Exclusion	Non-English language publication; non-peer-reviewed; book, dissertation, conference abstract; No primary data; quantitative-only studies; non-community-dwelling older adults; non-family member loss; non-human loss; no reference to grief/bereavement			
Time Frame	The scoping review was conducted in April 2024, our search was limited to studies published after 2000

**Table 2.** Data Extraction Template.

General Information	Study ID
	Author(s)
	Year of publication
	Country in which study conducted
	Objective of study
	Study design: Interviews; observations; focus groups; other
Characteristics of included studies: Methods	Sample size
	Age range
	Number of male participants
	Number of female participants
	Living arrangements
	Geographic characteristics (i.e., urban, rural, etc.)
	Other defining characteristics
	Limitations noted
Characteristics of included studies: Bereavement experiences	Type of loss: Spousal loss; parental loss; other family members; other
	Seek support: Yes; no; other
	Types of bereavement support sought: Grief specific organizations; professional counseling; informal social networks; online resources; no support sought; other
	Satisfaction of support
	Before COVID-19: Yes; no study occurred after March 2020
	If during COVID-19, implications to grief and support?
	Other comments on findings/discussion

From the selected databases (Table 1), literature was searched for articles published from 2000 to the date of the search commencement, with additional hand searching. This timeframe was selected because this field has significantly developed over the past two decades. Moreover, these databases were deemed appropriate for this review since they provide sources covering a wide array of subjects within social sciences, health, and aging research.

Predefined keywords focused on four key areas: older adults, community settings, bereavement, and qualitative and mixed methods. To be included, studies had to be written in English, focus on qualitative bereavement experiences of community-dwelling older adults who have lost a family member, and be published after 2000. Due to initial uncertainty about the volume of qualitative studies, mixed method studies were included if they featured verbatim transcripts. Exclusion criteria included studies without primary data, non-English language publications, quantitative-only studies (i.e., grief scales), non-community-dwelling older adults (i.e., living in institutional care settings, incarceration), grief experiences outside of the family or not involving humans (i.e., pet loss), or minimal reference to bereavement.

Search results were managed in Covidence and Zotero, where duplicates were identified and removed. First, all article titles and abstracts based on the inclusion and exclusion criteria were reviewed. Then, full texts were examined. Data extraction was organized by variables such as methods and bereavement characteristics (see Table 2; Appendix available by request).

This scoping review initially aimed to focus on older adults’ bereavement experiences during the COVID-19 pandemic, but was limited by insufficient empirical data. However, we analyzed how the pandemic influenced research trends in this field and compared these to pre-pandemic bereavement experiences. Only peer-reviewed literature was considered, while dissertations, non-peer-reviewed literature, books, grey literature, conference papers, and popular press were excluded to maintain a focus on peer-reviewed scholarly articles.

### Procedure

The screening process used Covidence, a web-based systematic and scoping review program designed to enhance the efficiency of evidence synthesis by guiding users through the scoping review steps. Relevant sources meeting inclusion criteria were imported into Covidence, and duplicates were removed. The first and third authors independently conducted two rounds of screening, beginning with a title and abstract review based on study eligibility criteria, discussing issues as they arose. The subsequent stage involved a full-text review, in which the authors assessed study eligibility criteria. Reasons for exclusion are detailed in a PRISMA flow diagram (Figure 1).

**Figure 1.**
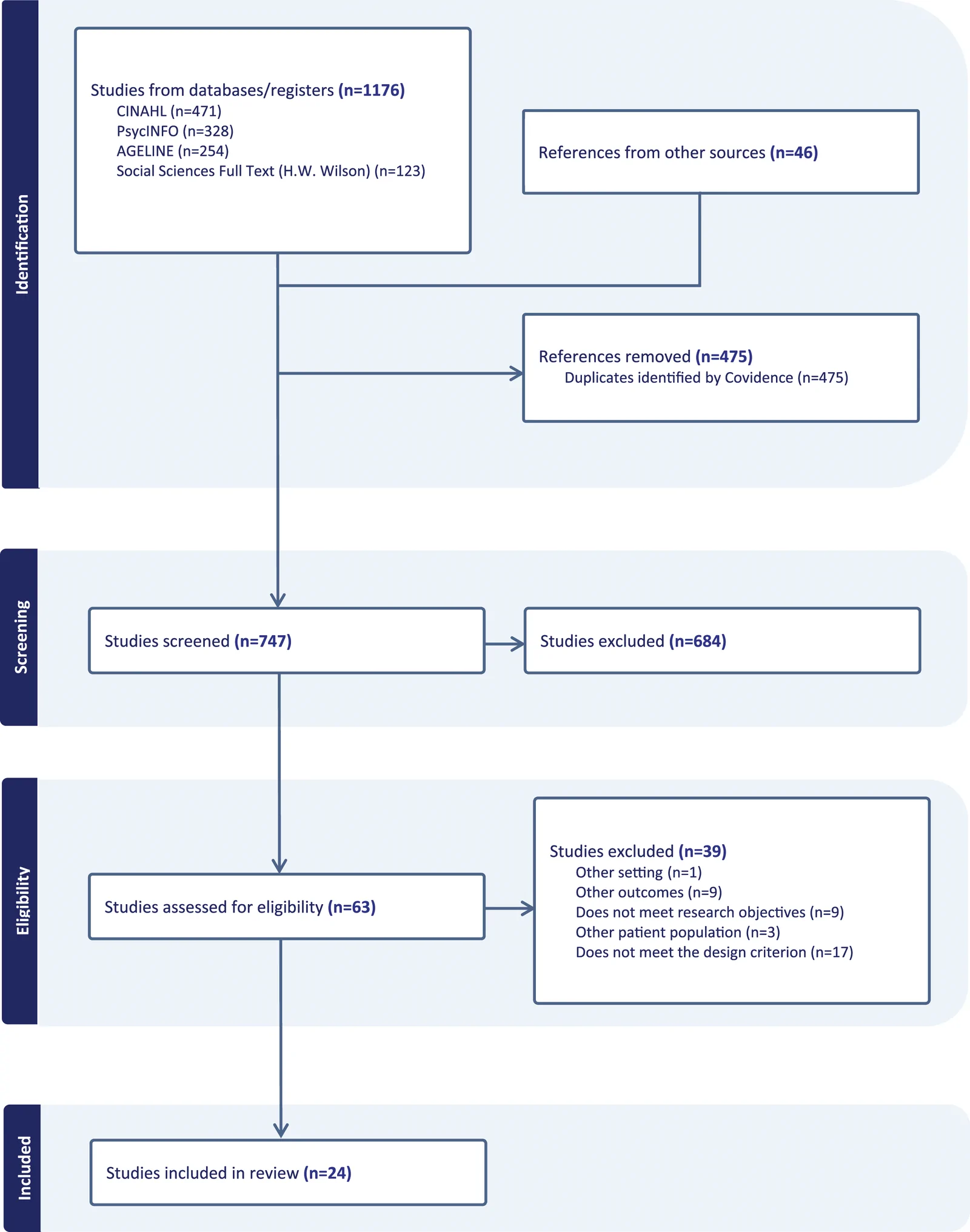
PRISMA flow diagram adapted from Covidence.

### Data Analysis

The first author extracted and organized key characteristics from the selected articles pertaining to community-dwelling older adults and bereavement. The extracted data were recorded in a custom Excel spreadsheet (refer to the Appendix). The second author cross-checked the extracted data with the first author to ensure alignment with the research objectives of all studies. Subsequently, a thematic analysis was undertaken to identify, analyze, and interpret patterns of meaning (‘themes’) across the included studies. This analytical approach proved valuable in generating patterns, common themes, topics, and ideas within and across the data, particularly regarding participants’ lived experiences, perspectives, and behavioral practices.

## Results

A total of 1176 studies were identified. After removing duplicates, 747 titles and abstracts were screened, with 684 studies excluded. The full-text review was conducted with 63 studies, 39 of which were excluded. The most common reasons for exclusion during the full-text review were that the article did not meet the design criterion (e.g., quantitative approaches with cross-sectional design), did not meet research objectives (e.g., grief experience was peripheral), other setting (e.g., prison), other outcomes (e.g., pet loss), and other patient population (e.g., healthcare staff perspectives). 24 studies were included based on all eligibility criteria.

### Characteristics of the Studies

An overview of the publication years and geographical context of the selected 24 articles can be found in Table 3. The majority of the studies were published after 2011. In terms of geographical context, majority of the studies were carried out in North America with representation from the United Kingdom, Australia, Europe, East Asia, the Middle East, and South Africa. It should be noted that two studies from Australia use the same dataset with two distinct focuses (i.e., financial wellbeing and health status).

**Table 3.** General Characteristics of Scoping Review.

Characteristics	Number (*n* = 24)	Percentage (%)
Publication year		
2000–2010	3	12.5
2011–2020	12	50
2021–2024	9	37.5
Geographical context		
North America	8	33.34
Europe & United Kingdom	7	29.17
East Asia & Middle East	4	16.67
Australia	4	16.67
South Africa	1	4.2

As well, some study research objectives did not primarily focus on bereavement or loss (*n* = 2), but rather on life events and meaning in life, where their experiences of widowhood and bereavement were foundational. Therefore, the first and second authors agreed that they would be eligible for inclusion.

The age of study participants ranged from 53-95. Five studies only included older women’s experiences of bereavement in their sample, and in only six studies the sample included equal or more males than females in the sample. Therefore, the selected studies highlight predominately female bereavement experiences. Only one study (e.g., Millette & Bourgeois-Guerin, 2020) included same-sex participants in their research design. Notably, living arrangements varied, with participants either living alone or with a spouse, family, or friends either in retirement communities or other community-dwelling settings.

An overview of the bereavement characteristics of the selected studies can be found in Table 4. The majority of the participants experienced spousal loss. The table also highlights that informal social support networks were described in most of the studies. Satisfaction of support was varied, including both positive and negative experiences, as well as the perception that grief is an individual issue. This discussion is expanded on in the next section. In most of the studies (*n* = 19), datasets were collected before the pandemic. In the five studies conducted during COVID-19, three observed its impact on accessing support and resources, one reported data collection issues, and one reported no discernible impact.

**Table 4.** Bereavement Characteristics of Scoping Review.

Characteristics	Number (*n* = 24)	Percentage (%)
Type of loss		
Spousal	21	87.5
Child	6	25
Sibling	3	12.5
Parent	7	29.17
Other family member	5	20.84
Type of support		
Informal social support networks	22	91.67
Professional counselling/therapy	5	20.84
Grief-specific organizations	3	12.5
Senior serving centers	4	16.67
Cultural and religious organizations	9	37.5
Recreational/volunteer groups	6	25
Online groups/forums	3	12.5
Other organizations (i.e., legal/financial aid, employment associations, healthcare programs)	10	41.67
Data collection occurred prior to COVID-19		
Yes	19	79.17
No	5	20.83

The first author employed a thematic coding approach (Gibbs, 2007) to identify patterns and themes, extracting key information relevant to the study. Subsequently, the data were organized and meticulously analyzed, resulting in the distillation of coded information into coherent and meaningful themes that captured the essence of the research findings. Through this thorough analysis of the content, four key themes were generated: (1) Impact of grief on wellbeing, (2) Grief perceived as an individual issue, (3) Challenges in seeking and receiving support, and (4) Positive support experiences and transformations.

### The Impact of Grief on Wellbeing

A central theme in most studies (*n* = 21) was the multifaceted impact of grief on the wellbeing of older adults, such as changes in financial stress (DiGiacomo et al., 2015; Shi, 2022; Statz et al., 2023), mental wellbeing (Holm et al., 2019; Hong et al., 2021; Hybholt et al., 2020; Nelson-Becker, 2004; Safa et al., 2021), spiritual wellbeing (Bui et al., 2018; Damianakis & Marziali, 2012; Lekalakala-Mokgele, 2018; Nelson-Becker, 2004; Safa et al., 2021), physical wellbeing and health behaviors (Bui et al., 2018; Digiacomo et al., 2013; Holm et al., 2019; Hong et al., 2021), and social wellbeing (Collins, 2018; Davidson, 2000; Fang & Carr, 2021; Gerber et al., 2022; Koren, 2016; Sopcheck, 2020). Many study participants faced new financial realities after their spouse’s death, sometimes leading to selling their home and relocating due to unexpected debts (DiGiacomo et al., 2015; Shi, 2022). Interactions with banks and financial advisors often exacerbated the emotional toll of grief (DiGiacomo et al., 2015). Some participants spoke to the gendered cohort effects of financial literacy and the challenges of managing finances independently after spousal death; “Like most women of my vintage, I didn’t have anything to do with the money” (DiGiacomo et al., 2015, p. 6). This participant, reflecting the experiences of others of this era, highlights a broader generational pattern where women may have been less involved in financial decision-making.

The impact on mental wellbeing is evident, with participants experiencing feelings of loneliness and depression, often affecting their sense of purpose and motivation (Collins, 2018; Gerber et al., 2022; Holm et al., 2019; Hybholt et al., 2020). In general, the impact on mental wellbeing varied across individual factors, including loss type, social support, health, and living arrangements. Critical to the bereavement experience in later life is how age intersects with grief. For many, later life is frequently marked by the compounding of multiple bereavement experiences, leading to an accumulation of loss and a decline in physical and mental wellbeing (Gerber et al., 2022; Lekalakala-Mokgele, 2018). In some cases, the psychological toll became so pronounced that individuals were prescribed antidepressants to cope. Initial resistance to the label of depression was countered by a desperate need for sleep (Digiacomo et al., 2013), illustrating the intricate relationship between mental and physical wellbeing.

While navigating bereavement, the decline in mental wellbeing can also trigger changes in health behaviours, including lapses in self-care leading to health issues, increased alcohol consumption, and changes in eating habits (Digiacomo et al., 2013; Holm et al., 2019; Sopcheck, 2020). Grief can also deeply impact spiritual wellbeing, often provoking unique opportunities for profound spiritual exploration (Damianakis & Marziali, 2012; Lekalakala-Mokgele, 2018; Nelson-Becker, 2004; Safa et al., 2021). For instance, participants shared personal stories of how spirituality helped them to reflect on their self-identity, life’s purpose, and their place in the world following spousal loss (Damianakis & Marziali, 2012). The role of spirituality and religious communities will be discussed further in the subsequent themes.

Grief profoundly affects social wellbeing in two ways: a loss of companionship and a broader decline in social networks. For older adults entering widowhood, this loss extends to the intimate connection they once shared with their spouse (Collins, 2018; Holm et al., 2019; Koren, 2016; Wang et al., 2023), creating a void that can be challenging to navigate. Secondly, grief not only affects individuals personally but also contributes to a broader decline in social networks after the death. The once robust support systems surrounding an individual may diminish, with friends and acquaintances withdrawing, unsure of how to offer comfort or support (Ang, 2023; Collins, 2018; Fang & Carr, 2021; Switsers et al., 2023). This decline in companionship emphasizes the profound impact of grief on social wellbeing, revealing how bereavement can erode vibrant social network ties, leaving individuals with a sense of isolation.

Experiences of declining physical wellbeing during bereavement include examples such as physical challenges of managing a property alone, loss of energy, non-active lifestyles, and difficulty sleeping (Bui et al., 2018; Costello & Kendrick, 2000; Digiacomo et al., 2013; Fang & Carr, 2021; Holm et al., 2019). To illustrate, one participant describes a weight change, “I am overweight at the moment and that’s because I’ve been very non-active. Usually, I go walking on the beach or something like that but for the last 6 months I haven’t” (Digiacomo et al., 2013, p. 4). Her experience highlights the intertwining of grief and physical wellbeing, emphasizing a recent lifestyle shift attributed to the past six months of emotional distress. Grief’s far-reaching effects are evident in this theme, highlighting its interconnected impact on emotional, physical, financial, spiritual, and social wellbeing.

### Grief Perceived as an Individual Issue

Throughout many articles (*n* = 13) was the perception that grief is viewed as an individual struggle, particularly among bereaved older adults (Ang, 2023; Collins, 2018; Damianakis & Marziali, 2012; Digiacomo et al., 2013; DiGiacomo et al., 2015; Fang & Carr, 2021; Holm et al., 2019; Hybholt et al., 2020; Koren, 2016; Millette & Bourgeois-Guerin, 2020; Shi, 2022; Wang et al., 2023). The overarching narrative suggests that individuals are compelled to manage their grief alone, contributing to a sense of isolation. Several factors, such as societal stigma and concerns about making others uncomfortable, appear to influence this self-managed approach. Wang et al.’s (2023) study participant, Lucy, noted difficulty in expressing her grief and emotions publicly. Her reluctance to disclose her struggles and inclination to appear resilient might be indicative of societal expectations surrounding grief, as she expressed, “No one else can solve your own personal experience” (Wang et al., 2023, p. 143). Lucy’s response may suggest a societal norm that values stoicism and restraint in the face of loss, discouraging individuals from acknowledging ongoing grief or seeking support. Relatedly, this perception of grief as an individual issue was closely associated with the notion of not wanting to burden family members and friends (Ang, 2023; Damianakis & Marziali, 2012; Holm et al., 2019; Wang et al., 2023). Those from cultures where death is taboo or mourning practices are minimalized, along with immigrants lacking an ethnocultural community, may encounter challenges in finding meaningful avenues to express and process their bereavement.

Many studies showed gender differences in expressing and managing grief (Ang, 2023; Collins, 2018; Davidson, 2000; Koren, 2016). Ang (2023) notes that the narratives of male participants were less expressive than women, remarking that they “felt uncomfortable drawing attention to [myself] from others” (p. 12982). Traditional societal expectations and norms regarding masculinity and femininity may dictate how grief is expressed and perceived, potentially stifling emotional expression. In terms of sexuality, older adults grieving the loss of their same-sex partner described this sense of isolation and grief as an individual issue, as a result of social stigma and discrimination (Millette & Bourgeois-Guerin, 2020). The grief experience within the LGBTQ + community is deeply influenced by societal attitudes, legal frameworks, and personal networks. As Millette and Bourgeois-Guerin (2020) describe, individuals grieving their same-sex partner may confront a societal landscape that does not fully recognize or validate the relationship or loss, resulting in disenfranchised grief and exacerbating feelings of isolation and alienation. As mentioned in earlier findings, cohort effects can significantly shape bereavement processes. Specifically, theoretical perspectives such as generational cohort theory offer insight into how generational attitudes are shaped by historical events and social changes, such as gender roles and expectations within the family that shape grief (DiGiacomo et al., 2015). As well, these approaches examine cohort and lifespan effects concerning the degree of stigma and legal recognition attached to sexuality during various sociohistorical timeframes (Millette & Bourgeois-Guerin, 2020). Overall, this theme underscores the individualized, and often solitary nature of grief, influenced by societal stigma, cultural norms, and personal beliefs.

### Challenges in Seeking and Receiving Support

The majority of the articles (*n* = 21) described challenges in seeking and receiving support during the bereavement process, encompassing practical challenges (DiGiacomo et al., 2015; Shi, 2022), cultural barriers (Shi, 2022; Wang et al., 2023), technological barriers (Hong et al., 2021), availability and quality of emotional support (Ang, 2023; Collins, 2018; Costello & Kendrick, 2000; Davidson, 2000; Gerber et al., 2022; Holm et al., 2019; Hybholt et al., 2020; Koren, 2016; Millette & Bourgeois-Guerin, 2020; Nelson-Becker, 2004; Safa et al., 2021; Sopcheck, 2020; Wang et al., 2023), and more recently, disruptions caused by the COVID-19 pandemic (Hong et al., 2021; Statz et al., 2023). As mentioned earlier, many practical tasks arise as a result of the death, some of which can induce financial strain. Bureaucratic aftermath challenges, such as dealing with banks and solicitors, family dynamics, and emotional tolls associated with tasks like transferring names and being the executor of a will reveal a gap in support systems (DiGiacomo et al., 2015; Shi, 2022). This process, while necessary, is often described as distressing. For instance, one participant remarked on the amount of personal and intimate details on her husband’s death certificate and the distribution of this information to banks and other personal accounts (i.e., PayPal) (DiGiacomo et al., 2015). This finding highlights the need for more consideration of the emotional wellbeing of grieving individuals and sensitivity in handling such documentation.

The availability and quality of emotional support can serve as significant barriers to older adults, as limited access to supportive networks or resources may leave them feeling isolated and alone in their grief (Ang, 2023; Bui et al., 2018; Fang & Carr, 2021; Gerber et al., 2022; Holm et al., 2019; Safa et al., 2021; Sopcheck, 2020). Additionally, inadequate support can exacerbate feelings of loneliness and hinder their ability to cope effectively (Ang, 2023; Fang & Carr, 2021; Gerber et al., 2022; Millette & Bourgeois-Guerin, 2020; Safa et al., 2021). Friends and family often provide significant support immediately following the death of someone significant, but this availability tends to decline over time. For example, in Gerber et al.’s (2022) study using poetry, Richard describes: “I had good support from family and friends, before the funeral, getting over all that stuff. But then, of course, all these people drift off and go home and you’re left on your own.” (p. 4). Richard’s experience captures the effects of changes in emotional support, leaving older adults grappling with their grief in increasingly solitary circumstances.

Additionally, mental health stigma within older adults may discourage them from seeking out or accepting support (Bui et al., 2018; Davidson, 2000; Holm et al., 2019; Wang et al., 2023), further compounding the barriers they face. Moreover, stigma or lack of empathy toward older adults’ grief can intersect with other forms of discrimination, such as ageism or sexism. Monique illustrates the challenges of grieving her same-sex partner: “It wasn’t what they said but the fact that they ignored it (…) Never talking about it (my loss), as if it (the relationship) had never existed (…), as if she hadn’t been my spouse” (Millette & Bourgeois-Guerin, 2020, p. 66). These challenges in obtaining support exacerbate feelings of disenfranchisement and complicate the grieving process. Relatedly, disregarding cultural identities can constitute as a form of discrimination that profoundly impacts grieving individuals. Cultural barriers impacting bereaved older adults’ support-seeking behaviours may include norms discouraging emotional expression either within or outside the family unit, language obstacles hindering communication and access to healthcare and social services, as well as differing cultural beliefs on death and mourning (Shi, 2022; Wang et al., 2023), which may result in disparities between available support and cultural needs.

It is critical to recognize how older adults have been disproportionately affected by the COVID-19 pandemic. In terms of bereavement experiences and support, the pandemic disrupted a sense of routine for older adults who are already struggling with grappling with a new reality (Statz et al., 2023). Access to healthcare, community organizations, and levels of social connectedness with friends and family were challenged due to changing physical distancing restrictions (Statz et al., 2023), all of which impact bereavement processes. For older adults, multiple experiences of bereavement were described as exceptionally challenging given the unprecedented circumstances. One study participant, Veronica, describes this difficult process: “I’m grieving the loss of my Mom, who died on March 6^th^, the loss of a friend who got COVID in a nursing home and the serious illness of a friend on a ventilator due to COVID who has a poor prognosis” (Statz et al., 2023, p. 2826). This quote vividly illustrates how Veronica is navigating multiple layers of grief, mourning the loss of her mother, grappling with the stark reality of the pandemic’s toll on vulnerable populations, and facing the uncertainty and fear for the future as another friend battles the illness on a ventilator. The convergence of multiple grief experiences, coupled with concerns of their own mortality, limited access to health and social services, and decreased participation in activities that promote social connectedness can result in prolonged isolation and grief for older adults (Statz et al., 2023), highlighting the urgent need for targeted interventions and support in times of crisis.

### Positive Support Experiences and Transformations

The final theme illuminates positive experiences and transformations in the lives of bereaved older adults when they receive support, both professionally and informally (*n* = 18). These experiences span social connectedness, identity reconstruction, and the formation and growth of social relations within institutions (i.e., healthcare settings, workplaces) (Bui et al., 2018; Digiacomo et al., 2013; Nelson-Becker, 2004), in virtual spaces (Hong et al., 2021; Hybholt et al., 2020), through community serving organizations (i.e., religious, cultural, grief-specific) (Ang, 2023; Bui et al., 2018; Collins, 2018; Damianakis & Marziali, 2012; Holm et al., 2019; Lekalakala-Mokgele, 2018; Nelson-Becker, 2004; Safa et al., 2021; Sopcheck, 2020; Switsers et al., 2023; Wang et al., 2023), and within personal social networks (Ang, 2023; Collins, 2018; Fang & Carr, 2021; Gerber et al., 2022; Holm et al., 2019; Hong et al., 2021; Hybholt et al., 2020; Koren, 2016; Millette & Bourgeois-Guerin, 2020; Sopcheck, 2020; Switsers et al., 2023; Wang et al., 2023). Following loss, individuals engage in meaning-making, a process by which “individuals reassess and revise their sense of how the world works after their worldviews have been challenged by loss,” thus involving identity reconstruction (Hieftje, 2012, p. 31 referencing Neimeyer & Anderson, 2002). Therefore, the bereaved, with the help of family, community, and professionals, will reconstruct their meaning, identity, and roles with the deceased. During this tumultuous period, older adults may seek others with similar experiences, such as structured support groups or informal gatherings, which provide a safe space to share stories, exchange coping strategies, and draw strength from the collective resilience of the group. Formal grief support, like counselling, can provide a therapeutic space for discussing loss and aiding identity reconstruction (Bui et al., 2018; Damianakis & Marziali, 2012; Digiacomo et al., 2013; Hybholt et al., 2020; Sopcheck, 2020; Wang et al., 2023). As described by one widow, “It permits you to talk about your husband and his death and dying and I know it sounds morbid, but I think it helps to get it out. I can talk about it over and over and I think it does me good” (Digiacomo et al., 2013, p. 7).

In the face of loss, many individuals turn to their culture, religion, and spirituality for guidance, drawing upon their traditions, rituals, and beliefs to navigate their grief (Ang, 2023; Bui et al., 2018; Lekalakala-Mokgele, 2018; Nelson-Becker, 2004; Safa et al., 2021; Sopcheck, 2020; Switsers et al., 2023; Wang et al., 2023). For older adults, turning to culture, religion, and spirituality after loss may be particularly important as these aspects often serve as pillars of their identity and sources of comfort throughout their lives. Other experiences within the community include the role of targeted interventions of support such as Alzheimer’s associations, senior serving centers, and living environments such as retirement communities, which can become essential sources of social support to mitigate loneliness in later life. For many participants, community centers were important in mitigating loneliness in their bereavement (Collins, 2018; Fang & Carr, 2021; Sopcheck, 2020).

In recent years technological interventions have become valuable tools in supporting grieving older adults. With adequate digital literacy skills, virtual support groups, and online communities can connect those who share similar experiences, regardless of geographical constraints. In one study, a participant describes the comfort she receives through video chats with her friends and family (Hong et al., 2021). This sentiment is echoed in another study where the participant discusses a Facebook memorial for their son, “You can see those football pictures from the matches which is EXACTLY when he was happy. […] I like seeing the pictures where he… him in the football stadium (tearful voice)” (Hybholt et al., 2020, pp. 5–6). These examples highlight how technological interventions can offer grieving older adults a sense of emotional comfort and connection. Online tools can help maintain bonds with family, friends, and the deceased. However, it’s essential to note that digital literacy varies among older adults.

Friends and family were described as important sources of support, by providing a sense of companionship, understanding, and empathy during times of loss, helping to alleviate feelings of loneliness and isolation (Ang, 2023; Collins, 2018; Holm et al., 2019; Hybholt et al., 2020; Sopcheck, 2020; Switsers et al., 2023). Additionally, friends and family members can offer practical assistance with daily tasks, provide a listening ear, and offer validation and reassurance (Collins, 2018; Nelson-Becker, 2004; Sopcheck, 2020; Switsers et al., 2023; Wang et al., 2023). Maintaining a connection with the deceased can serve as a form of ongoing support for grieving older adults, as supported by continuing bonds theorizations (Klass et al., 1996). Activities that honour the memory of the deceased, such as creating memorial keepsakes, visiting their favourite places, or celebrating special occasions in their honour, allows older adults to preserve the relationship and legacy (Ang, 2023; Costello & Kendrick, 2000; Fang & Carr, 2021; Holm et al., 2019; Hybholt et al., 2020). As one widower described, “I talk to my wife every day and I discuss my problems with her, she is the best person to speak to because she is my best friend and understands me more than anyone else in the world” (Costello & Kendrick, 2000, p. 1379). These ongoing interactions not only provide comfort and support but also reinforce the enduring bond between the grieving individual and the deceased, offering a sense of continuity and connection that transcends physical absence. This theme underscores the positive outcomes and transformations that bereaved older adults can experience with comprehensive support. It emphasizes social connectedness, identity reconstruction, and the formation and growth of social relations as crucial elements in the grieving process.

## Discussion

The four themes identified through this review offer rich and nuanced insights into the multifaceted nature of bereavement experiences in later life. Firstly, the recognition of grief’s profound impact on wellbeing highlights the intricate interplay between loss and various dimensions of health. For older adults, the intertwining of grief with physical health can exacerbate existing health conditions as well as potentially contribute to the development of new health issues (Fagundes & Wu, 2020). Thus, it is essential to acknowledge that grief can permeate all aspects of wellbeing, triggering a stress response leading to physiological effects on the body, while also increasing the likelihood of engaging in unhealthy coping behaviours. To illustrate, one notable risk factor linked to substance abuse in later life stems from challenges associated with coping with grief and loss (Chasek, 2022). Lack of social support and meaningful social connections can contribute to feelings of loneliness and depression, which in turn can impact physical wellbeing and mortality risk (Holt-Lunstad et al., 2015; Tzeng et al., 2023). Social support can serve as a protective factor against the detrimental effects of grief and loss (Chen, 2022; Nam, 2016). Thus, implementing community care frameworks is critical to the wellbeing of grieving older adults and social connectedness within community contexts, an argument that will be developed further in the next theme.

The recognition of grief’s profound impact on wellbeing also underscores the necessity of considering social determinants of health approaches (Raphael, 2016), recognizing how various modes of identity, such as health status, accessibility, and socioeconomic status shape grief experiences. These factors may disproportionately affect individuals, amplifying the complexity of their bereavement experiences, highlighting the need for tailored support interventions that address these intersecting dimensions of identity and circumstance. These interventions would not only help people navigate their grief, but they may also save the bereaved from a trajectory into being unhoused (Monk et al., 2023).

Second, perceiving grief as an individual issue underscores the subjective nature of bereavement experiences. Each person’s journey through grief is shaped by a myriad of factors, including their unique relationship with the deceased, cultural background, past experiences with loss, health status, availability of social support, and individual coping mechanisms (Ott et al., 2007; Schmall & Bowman, 1993). While commonalities may emerge among older adults experiencing loss, each person’s grief experience is deeply personal and may manifest in ways that are unique to their circumstances and life stage. This understanding prompts a departure from generalized assumptions about how older adults should grieve, such as harmful assumptions about the ‘timeliness’ of grief experiences in later life (Schmall & Bowman, 1993), and calls for a more nuanced approach that acknowledges and accommodates individual variation and expression within the community context. This insight invites consideration of how grief intersects with other aspects of older adults’ lives, such as their sense of identity, social connections, and broader existential questions about aging and mortality. Examining the role of structural vulnerability due to social disparities, such as outcomes like social disenfranchisement and psychological distress, highlights areas needing practice and policy improvements (Bindley et al., 2019). The importance given to personal experience might be influenced by methodological factors, as eligibility criteria for the selected studies in this review were in English. With only two studies conducted in Asian contexts, it’s worth considering how research conducted in other cultures and languages could offer varied insights into how collectivist cultures shape grief experiences. In collectivist cultures – where the emphasis is on interdependence, communal support, and group cohesion – solace and meaning may be found in shared rituals, collective mourning practices and the support inherent in the broader community and family structure (Walter, 2010). Consequently, this theme may not necessarily apply or resonate within collectivist cultures.

Moreover, as we acknowledge grief as a deeply personal experience within the community, it becomes imperative to adopt community care frameworks, such as the “compassionate communities” approach (Kellehear, 2005) that prioritizes community engagement and support in addressing death, dying, and bereavement. In a compassionate community, illness, wellbeing, birth, death, love, and grief are integral to community life, involving institutions in recognizing death and grief as part of their responsibilities (Kellehear, 2005). Reflecting on the earlier discussion concerning the role of banks and financial institutions and their impact on grief experiences of their clientele, it becomes pivotal to appreciate the importance of community care frameworks, exemplified by Kellehear’s approach. Thus, for community-dwelling older adults the compassionate communities framework offers a shift in perspective from viewing death as solely a medical event and grief as an individual issue, to understanding it as a process that occurs within the context of family and community. By incorporating the principles of compassionate communities into bereavement support, social services and broader community initiatives, this shift in perspective can empower older adults to actively seek and access support from their community networks.

Third, identifying challenges in seeking and receiving support reveals systemic barriers that hinder access to essential services, advocating for improved accessibility and awareness in community settings. As described in several studies (e.g., Wang et al., 2023), these barriers may include limited availability of grief support resources, lack of awareness about existing services, stigma surrounding grief and mental health issues, as well as logistical challenges such as transportation or mobility issues. This involves not only addressing structural constraints but also promoting a culture of openness and acceptance regarding grief and mental health issues among community members and service providers. To address these barriers, community leaders, healthcare professionals, and local organizations can collaborate with bereavement service providers to develop and implement initiatives that increase access to bereavement resources for older adults. For instance, providers in a hospital-based mind-body program for grieving older adults expressed a desire to extend similar programs to communities: “Our volunteers would eat this up and love to do it, and if they were doing it with a clinician, I think that the room would be full.” (Bui et al., 2018, p. 863). Ensuring that bereavement support services are culturally sensitive and inclusive is essential, such as providing services in multiple languages, incorporating cultural traditions and practices into support programs, and engaging with community leaders and older adults to ensure that services are respectful of cultural norms and values (Mayland et al., 2021). In particular, the growing body of literature on Indigenous bereavement raises critical debates about the need to “decolonize bereavement” (Hamilton et al., 2022) and grapple with the intricate colonial histories influencing bereavement studies and practices. Addressing these barriers will not only foster resilience among grieving older adults but additionally, will strengthen social cohesion within the community.

Lastly, exploring positive support experiences and transformations illuminates the potential for growth and resilience amidst loss, emphasizing the importance of nurturing supportive communities. Positive support experiences may include instances where older adults receive empathy, companionship, and practical assistance from friends, family, neighbours, or community organizations following the loss of someone significant. These acts of kindness and support provide immediate comfort and contribute to a sense of security and reassurance during a vulnerable time. Moreover, amidst grief, older adults may undergo personal growth and transformation, discovering new strengths, insights, and perspectives on life. This process of post-traumatic growth may involve finding renewed purpose or meaning, deepening relationships with others, or developing greater resilience and coping skills in the face of adversity (Calhoun et al., 2010). For community-dwelling older adults, cultivating environments that promote feelings of value, understanding, and support can create spaces for sharing experiences, expressing emotions, and receiving the necessary care and support to navigate grief. Additionally, supportive communities can provide opportunities for older adults to engage in meaningful volunteer activities and social interaction which can contribute to a sense of purpose and fulfillment in later life (Onyx & Warburton, 2003). By fostering a sense of belonging and connection, communities can help older adults build and maintain social networks that serve as sources of support and strength during times of loss and transition.

Critical to the conceptual significance of these themes is their interconnectedness. The recognition of grief’s impact on wellbeing informs the perception of grief as an individualized issue, acknowledging the diverse coping mechanisms individuals employ. Challenges in seeking and receiving support stem from this individualized perspective, highlighting barriers that hinder access to necessary supports. Conversely, positive support experiences and transformations demonstrate the potential for growth and resilience when adequate support is available, underscoring the importance of addressing barriers and fostering supportive communities.

### Practical Implications

The identified themes highlight key implications for bereavement support and intervention. These recommendations include acknowledging how societal, systemic, and individual factors shape bereavement experiences in the community. Given the profound impact of grief on wellbeing, a holistic, tailored approach is essential to address individual variations in coping and expressions of grief. Systemic barriers, such as limited access to support services, highlight the need for initiatives like the Collaborative Action Plan in British Columbia (BC Centre for Palliative Care, 2023) to improve accessiblity and awareness. Furthermore, addressing program issues related to urban/rural discrepancies could involve implementing mobile bereavement support units or telehealth services to reach individuals in remote areas, ensuring equitable access to grief support resources.

Societal factors often shape the perception of grief as an individual struggle, influenced by norms and generational attitudes surrounding death, grief, mental health and psychotherapy, which often value stoicism and self-reliance (Brownlie, 2009; Hannaford et al., 2019; Walter, 2017). These attitudes can lead to isolation and limited support systems, especially for older adults, who may be facing shrinking social networks, gaps in financial and digital literacy, as well as disparities in household responsibilities. Community-based interventions, guided by social gerontology, can help to counteract isolation and bolster support networks. Societal attitudes and stigma also play a role in the reluctance to accept services, highlighting the need for relevant institutions and organizations to acknowledge and support grieving individuals ethically. Strengthening community ties, in conjunction with collaborative efforts involving bereavement service providers, healthcare and social services, through community-level organizations tailored to specific needs and populations (i.e., cultural and religious associations, senior-serving organizations, etc.) can provide essential support. Relatedly, these supports should integrate concepts such as grief literacy (Breen et al., 2020) into their programs as a way to promote open communication and supportive relationships. Grief literacy increases awareness and knowledge about the diverse ways grief can manifest in later life and enable community members to provide more informed and supportive responses to grieving older adults, fostering greater empathy, compassion, and connection.

### Limitations

While this review provides valuable insights into the qualitative experiences of bereavement, only peer-reviewed articles were included. This decision meant excluding books and grey literature such as reports and conference papers, which might have provided additional insights. As well, the focus on English-language studies may have inadvertently excluded valuable research from non-English speaking contexts. Relatedly, geographical representation was also limited, with most studies from North America, Europe, and Australia, potentially reducing the findings’ global applicability. Furthermore, the review could benefit from exploring the intersection of grief and other social factors such as socioeconomic status and access to healthcare. While these factors were not consistently described in the studies included in this review, they remain crucial for understanding the full complexity of bereavement. Future research, particularly a broader review that encompasses both community-dwelling and institutional experiences, would allow for an in-depth examination of how social determinants of health shape the bereavement experience.

### Future Research

Longitudinal studies can track bereavement trajectories over time and provide insights into the fluidity of grief, though they are limited by participant attrition, particularly when emotional distress or life changes hinder consistent participation. Retention of older participants presents another challenge, due to factors such as declining health or mortality that may disrupt data continuity. Further examination of socio-cultural processes and multicultural perspectives also holds significant potential for uncovering the varied coping strategies employed by diverse populations in response to loss. Future research needs to prioritize the examination of Indigenous grief and bereavement, with a particular focus on understanding the legacy of residential schools and cycles of intergenerational trauma. Moreover, investigating the role of technology and digital support mechanisms is crucial. This includes exploring various technologies, such as grief support websites or mobile applications. As Lowe et al. (2021) note, memorialization practices are changing, with bereaved individuals seeking more personalized and informal ways to honour the deceased. Future research should evaluate the effectiveness of community-based interventions tailored to specific needs, such as outreach programs and grief-specific support groups (i.e., pet loss), as a critical area of inquiry for understanding the changing landscape of bereavement support practices.

The COVID-19 pandemic’s impact on bereavement experiences, including aspects such as unresolved grief and ‘pandemic grief’ (Ummel et al., 2022), requires a deeper examination of which will enable us to grasp the interplay between public health crises and individual mourning processes. Relatedly, examining hybrid support practices that blend in-person interventions with technology is critical for assessing their accessibility and effectiveness in bereavement care, and long-term studies can uncover adjustment patterns and inform improvements to these approaches. Mueller et al. (2023) provide insight into interventions designed to support the bereaved the COVID-19 era, highlighting the critical need for adaptive support systems during crises. Furthermore, MacNeil et al. (2023) explore the use of virtual funerals during the pandemic, contributing to a growing body of work that examines how technological solutions can address the constraints imposed by public health measures. Both studies underscore the importance of balancing accessibility with the emotional and cultural needs of mourners, yet further research is needed on the unique bereavement experience of older adults during this unprecedented time. In particular, research is needed to address ethical considerations in bereavement care during times of crisis, including equitable access, cultural sensitivity, and virtual settings.

In conclusion, this scoping review offers a timely and comprehensive exploration of the multifaceted landscape of bereavement experiences and support among community-dwelling older adults. The interconnectedness of these themes underscores the complex interplay between individual, societal, and systemic factors in shaping grief processes and tailored support needs. By synthesizing existing literature and identifying gaps in knowledge, this scoping review serves as a valuable resource for informing future research directions and guiding the development of effective interventions to support older adults navigating bereavement in their communities.

## Supplemental Material

Supplemental Material - Qualitative Bereavement Experiences and Support in Community-Dwelling Older Adults: A Scoping Review Supplemental Material for Qualitative Bereavement Experiences and Support in Community-Dwelling Older Adults: A Scoping Review by Samantha Teichman, Barbara A. Mitchell, Indira Riadi, Habib Chaudhury, Albert Banerjee, and William Odom in OMEGA - Journal of Death and Dying.
